# Capillary origami: superhydrophobic ribbon surfaces and liquid marbles

**DOI:** 10.3762/bjnano.2.18

**Published:** 2011-03-10

**Authors:** Glen McHale, Michael I Newton, Neil J Shirtcliffe, Nicasio R Geraldi

**Affiliations:** 1 School of Science and Technology, Nottingham Trent University, Clifton Lane, Nottingham NG11 8NS, UK

**Keywords:** capillary origami, Cassie, contact angle, superhydrophobic, Wenzel

## Abstract

In the wetting of a solid by a liquid it is often assumed that the substrate is rigid. However, for an elastic substrate the rigidity depends on the cube of its thickness and so reduces rapidly as the substrate becomes thinner as it approaches becoming a thin sheet. In such circumstances, it has been shown that the capillary forces caused by a contacting droplet of a liquid can shape the solid rather than the solid shaping the liquid. A substrate can be bent and folded as a (pinned) droplet evaporates or even instantaneously and spontaneously wrapped on contact with a droplet. When this effect is used to create three dimensional shapes from initially flat sheets, the effect is called *capillary origami* or *droplet wrapping*.

In this work, we consider how the conditions for the spontaneous, capillary induced, folding of a thin ribbon substrate might be altered by a rigid surface structure that, for a rigid substrate, would be expected to create Cassie–Baxter and Wenzel effects. For smooth thin substrates, droplet wrapping can occur for all liquids, including those for which the Young’s law contact angle (defined by the interfacial tensions) is greater than 90° and which would therefore normally be considered relatively hydrophobic. However, consideration of the balance between bending and interfacial energies suggests that the tendency for droplet wrapping can be suppressed for some liquids by providing the flexible solid surface with a rigid topographic structure. In general, it is known that when a liquid interacts with such a structure it can either fully penetrate the structure (the Wenzel case) or it can bridge between the asperities of the structure (the Cassie–Baxter case).

In this report, we show theoretically that droplet wrapping should occur with both types of solid–liquid contact. We also derive a condition for the transition between the Cassie–Baxter and Wenzel type droplet wrapping and relate it to the same transition condition known to apply to superhydrophobic surfaces. The results are given for both droplets being wrapped by thin ribbons and for solid grains encapsulating droplets to form liquid marbles.

## Introduction

In wetting, the usual implicit assumption is that a solid substrate is sufficiently thick or rigid, that it does not deform or change shape due to the interfacial forces that arise when it contacts a droplet of a liquid, however, in many natural systems this is not the case. Depositing a small droplet onto a smooth substrate and measuring the contact angle in side-profile view gives the contact angle, θ, which is assumed (to within contact angle hysteresis) to approximate to the Young’s law value, θ_e_, given by the interfacial tensions, i.e., cosθ_e_ = (γ_SV_ − γ_SL_)/γ_LV_ where the γ_ij_ are the interfacial tensions between the solid, liquid and vapor phases. However, the bending rigidity of a solid elastic plate scales with the cube of its thickness and this assumption can become erroneous [1]. When a droplet has a radius, *R*, larger than the elastocapillary bending length [2], *L*_EC_ = (κ_b_/γ_LV_)^1/2^ the solid can become deformed and shaped by the liquid. In practice, this effect has been given the name “*capillary origami*” based on experiments showing how films of polydimethylsiloxane (PDMS) shaped in two-dimensions can be folded by evaporating droplets of water to produce a designed three-dimensional shape [3–4]; an effect stronger than the dimpling of an elastomer surface by a deposited droplet [5]. *Capillary origami* is more than a curiosity and has implications for technological applications in creating three-dimensional structures from initially flat films through the capillary forces during liquid evaporation and drying [6–8]. The effect of capillary forces due to nanodroplets in activating and guiding the folding of planar graphene ribbons has recently been simulated [9].

**Figure 1:**
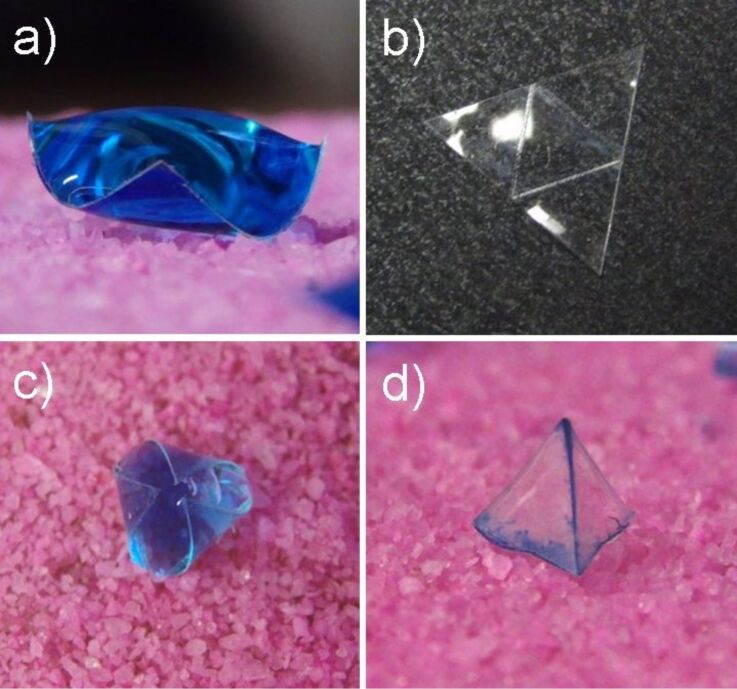
Effect of droplets of blue-dyed water on a thin polydimethylsiloxane (PDMS) membrane: a) droplet causing bending of the substrate, b) initial shaped substrate with the three score lines for folding, c) droplet induced folding, and d) three-dimensional shape left after completion of evaporation.

Figure 1 illustrates *capillary origami* concepts and effects based on original ideas by Py et al [3–4]. When a PDMS (Sylgard 184) substrate of reduced thickness is contacted by a droplet of water (containing blue food dye) capillary forces bend it out of its initial planar shape (Figure 1a). When the substrate thickness is reduced to 45 μm and cut into a triangular shape (10 mm side lengths) and scored with a laser (Universal Laser Systems 30W CO_2_ laser cutter) to create fold-lines (Figure 1b), contact with a large droplet of water can create a three-dimensional shape (Figure 1d). On contact by the droplet the sheet is bent (Figure 1c) and after droplet evaporation a tetrahedron is formed (Figure 1d). Whilst this is an example of the shaping of a solid substrate by capillary forces, the final shape relies on evaporation to complete the process.

**Figure 2:**
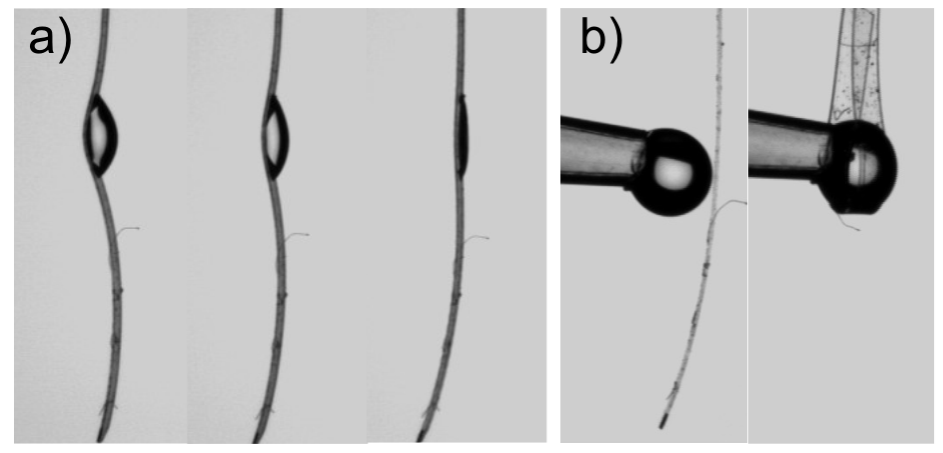
Effect of droplets of water on a thin polydimethylsiloxane (PDMS) membrane ribbon substrate hanging vertically: a) droplet causing a bending of the substrate which disappears as evaporation proceeds (three frames), b) spontaneous wrapping as a droplet touches a membrane ribbon (initial and final states).

Figure 2 illustrates a number of effects as a droplet contacts a thin PDMS strip substrate (“ribbon”) hanging vertically. If a droplet is deposited on a long ribbon it causes substrate deformation, but is unable to wrap or fold the substrate around itself and, as evaporation proceeds, the deformation decreases (Figure 2a). However, when the length of ribbon below the droplet contact point is sufficiently short, the contacting droplet can quickly fold the ribbon up against gravity and wrap itself. Figure 1 and Figure 2 are illustrative of the ability of capillary forces to deform, fold and bend substrates. The concepts of *capillary origami* and *droplet wrapping* also have implications for our understanding of the definition of hydrophobicity and its relationship to adhesion. Gao and McCarthy demonstrated that spontaneous and complete droplet wrapping occurs, without the need for evaporation, with a thin film of Teflon® even though this material would normally have a contact angle to water greater than 90° and so be regarded as hydrophobic [10]; an effect one of the current authors explained on the basis of the changes in the balance between interfacial and bending energies [11].

In a previous report, McHale argued from surface free energy considerations that, when the bending energy is small, all solids should demonstrate droplet wrapping and so can, in an absolute sense, be considered hydrophilic [11]. That work also discussed why for a partially wetting droplet to be observed there is necessarily an assumption of some rigidity of the substrate, so that the usual definition of relative hydrophobicity (and relative hydrophilicity) through contact angle measurement includes a structural non-surface chemistry based assumption about the solid. It was also suggested that a set of loose spherical grains could be considered to be the extreme case of a solid with no bending energy, thus relating the concept of droplet wrapping to that of the formation of liquid marbles [12–13]. It was further argued that when the flexible solid surface possessed rigid surface roughness or the solid grains had a rigid surface roughness, droplet wrapping might, under defined conditions for the surface chemistry defined contact angle, be suppressed. Since wrapping a spherical droplet requires both bending and stretching of the solid, in this report, we consider the simpler, but experimentally realizable, cases of wrapping of a droplet of water by a thin ribbon and the assembly of solid grains to form a liquid marble. For both cases, we extend the previous theoretical consideration to ribbon-type substrates and disconnected solid grains with a rigid surface structure. We review the case for surface roughness that has low aspect ratio so that the liquid can penetrate into the structure – the Wenzel case [14–15]. We then consider whether droplet wrapping can occur without penetration into the surface structure – the Cassie–Baxter case [16–17]. We show that droplet wrapping should occur with both types of configuration and we derive a condition for the transition between these two cases; this condition is the same as for the Wenzel to Cassie–Baxter transition on a superhydrophobic surface [18–19].

## Results and Discussion

### 1. Droplet wrapping theory

**Figure 3:**
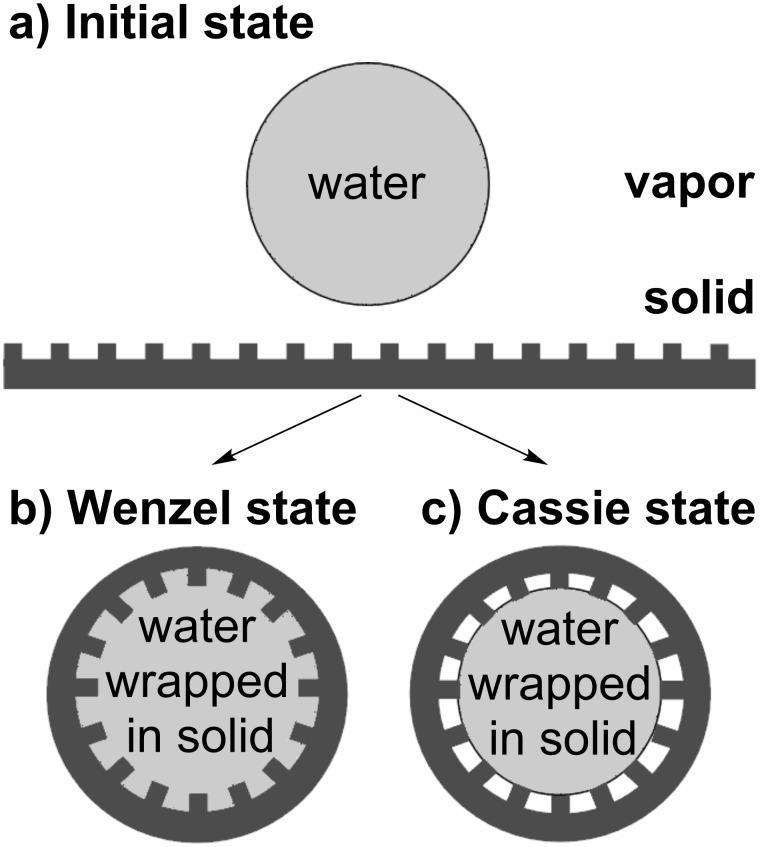
Initial and final states involved in a droplet wrapping event for a flexible ribbon membrane with rigid roughness. In the Wenzel case the liquid penetrates between features and in the Cassie case it bridges between them.

To assess whether it is energetically favourable for a liquid to become wrapped in a solid we consider the change in interfacial energy as the solid–vapor interface is replaced by a solid–liquid interface together with the increase in bending energy as the solid deforms from a planar ribbon, similar to those shown in Figure 2, of width *w* << *R*, where *R* is the droplet radius. The use of a ribbon substrate allows the problem to be simplified to a quasi-two dimensional situation. Assuming there is no spontaneous curvature of the solid film, the initial energy is given by the sum of the energy associated with the liquid in contact with the vapor and the surfaces of the solid in contact with the vapor (Figure 3a),

[1]
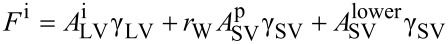


where *A*^i^_LV_ is the initial liquid–vapor interfacial area, *A*^p^_SV_ is the initial planar projection of the area of the upper surface of the solid film, *r*_W_ is the Wenzel roughness of the surface, and the γ_ij_ are the interfacial tensions; the lower surface of the film is assumed to have an area *A*^lower^_SV_. The initial liquid-vapor area is *A*^i^_LV_ = 4π*R*^2^, where *R* is the droplet radius, and after wrapping it is assumed that the shape is spherical with the same radius *R*. This means that a planar projected area 2π*Rw* of the ribbon’s area is involved in the wrapping. For simplicity in the following, we limit the initial ribbon length to 2π*R*, so that *A*^p^_SV_ = 2π*Rw* is assumed.

The energy per unit area, *f*_b_, associated with bending and stretching a thin membrane substrate is related to the principal radii of curvatures of the substrate,

[2]
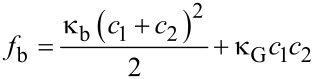


where κ_b_ is the elastic bending rigidity and κ_G_ is the Gaussian bending modulus [20]. For a film of thickness *h*, the bending rigidity is given by κ_b_ = *Eh*^3^/12(1−*ν*^2^), where *E* is Young’s modulus and *ν* is Poisson’s ratio; the Gaussian bending modulus relates to any stretching or compression of the film. The coefficients *c*_1_ and *c*_2_ are the principal radii of curvature, which for a spherical droplet are *c*_1_ = *c*_2_ = 1/*R*. For a ribbon bending only along its length *c*_1_ = 1/*R* and *c*_2_ = 0 so that for a radius of *R* the bending energy per unit area is,

[3]
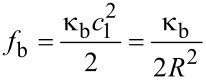


When the liquid comes into contact with the ribbon, assuming the ribbon can bend, and that the roughness remains unchanged, we can imagine two types of wrapping scenarios. In the Wenzel case, the liquid may penetrate between the surface features and retain contact with the ribbon at all points along its surface (Figure 3b). In the alternative Cassie–Baxter case, the surface structure combined with the surface chemistry may be such that the liquid bridges between the tops of the surface features leaving vapor between them (Figure 3c).

#### 1.1 Wenzel case

In the Wenzel case, the liquid penetrates between surface features (Figure 3b) and the difference in energy between the final and the initial state related to the attachment of the droplet to the ribbon is given by,

[4]
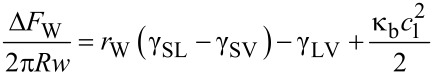


which can be rewritten using the definition of the Young’s law equilibrium contact angle on a rigid surface of cosθ_e_ = (γ_SV_ − γ_SL_)/γ_LV_, as,

[5]
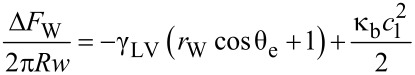


For liquids which on a rigid smooth solid substrate are considered to be partially wetting the cosine satisfies −1 < cosθ_e_ < 1 and θ_e_ gives a finite Young’s law contact angle. However, for those liquids which completely wet and form films, the combination (γ_SV_ − γ_SL_)/γ_LV_ has a value greater than 1. The combination of the roughness, *r*_W_, multiplying cosθ_e_ immediately introduces the Wenzel contact angle,

[6]
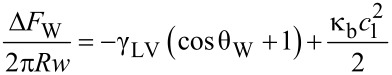


One assumption in Equation 6 is that the final radius of the wrapped portion of the droplet is approximately the same as the initial droplet radius.

#### 1.2 Cassie–Baxter case

In the Cassie–Baxter case, complete penetration of liquid between surface features does not occur (Figure 3c). The liquid only contacts a fraction φ_s_ of the surface thus leaving a fraction (*r*_W_ − φ_s_) of the solid surface in contact with the vapor. In addition, the liquid bridges between surface features, thus providing a set of menisci, here approximated by a fraction (1 − φ_s_) of the surface with a liquid–vapor interface. The difference in energy between the final and the initial state related to the attachment of the droplet to the ribbon is then given by,

[7]
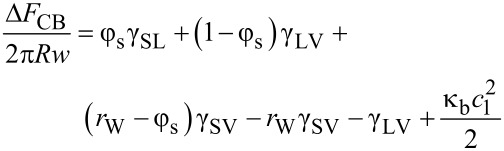


Cancelling terms involving the roughness factor *r*_W_ and using the definition of the equilibrium contact angle on a rigid substrate of cosθ_e_ = (γ_SV_ − γ_SL_)/γ_LV_ gives,

[8]
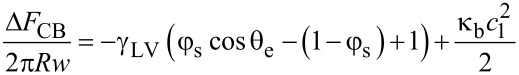


Defining the Cassie–Baxter combination cosθ_CB_ = φ_s_cosθ_e_ − (1−φ_s_), which is familiar from the modelling of droplets on superhydrophobic surfaces, gives,

[9]
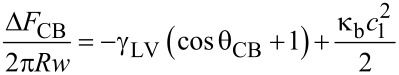


The similarity of Equation 6 and Equation 9 can be revealed by writing,

[10]
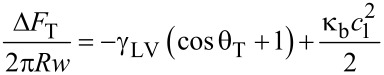


where the subscript T defines the topographic assumption of the liquid either in a Wenzel (“penetrating”) or Cassie–Baxter (“skating”) state. In the form presented by Equation 10, the principal radius of curvature *c*_1_ is given by 1/*R* and so the energy change per unit area of the ribbon substrate depends on the droplet size.

#### 1.3 Wrapping and transitions with roughness

The wrapping state will be stable provided the energy change given by Equation 10 is negative, i.e.,

[11]
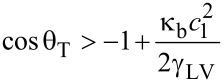


Defining the dimensionless curvature elastocapillary number *n*_EC_ = κ_b_*c*_1_^2^/2γ_LV_, Equation 11 can be written as,

[12]
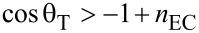


A ribbon substrate that is unable to bend in response to contact with the liquid will have an elastocapillary number that tends to infinity and so wrapping will not occur. When the elastocapillary number has a finite value, wrapping will occur, but will depend on the volume and shape of the liquid. For a droplet with a spherical shape of radius *R*, the elastocapillary number is *n*_EC_ = *κ*_b_/2γ_LV_*R*^2^ = ½(*L*_EC_/*R*)^2^, where *L*_EC_ = (*κ*_b_/γ_LV_)^1/2^ is the characteristic elastocapillary length. Equation 12 then becomes,

[13]
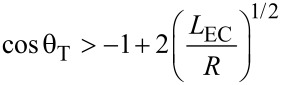


This condition for droplet wrapping depends upon the state of contact of the wrapped liquid with the solid surface, i.e., penetrating or skating. For the Cassie–Baxter state with its air-pockets to be thermodynamically stable compared to the Wenzel state, requires Δ*F*_CB_ < Δ*F*_W_ in addition to Δ*F*_CB_ < 0. Since the curvature energy contributes the same to both, Equation 10 implies cosθ_W_ < cosθ_CB_, which gives a condition on the relationship between the Young’s law contact angle θ_e_, and the roughness *r*_W_ and solid surface fraction φ_s_,

[14]
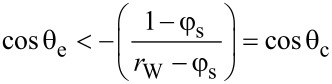


where θ_c_ is a critical contact angle for thermodynamic stability of the Cassie–Baxter state; when the Young’s law contact angle exceeds the critical contact angle the Cassie–Baxter state is favoured over the Wenzel state. Equation 14 is exactly the same as the condition derived by Bico et al*.*, for the thermodynamic stability of the Cassie–Baxter state on a superhydrophobic surface [18–19]. As noted by these authors, when 90° < θ_e_ < θ_c_, the Cassie–Baxter state may exist due to, e.g., pinning on sharp edges of features, but it is a metastable state.

Here we have also only considered a simple model that assumes either a Wenzel state or a Cassie–Baxter state. However, surfaces with curvature can effectively have a combination of both Wenzel and Cassie–Baxter properties with the solid surface fraction becoming a function of the Young’s law contact angle [21]. Re-entrant surfaces have been shown to be particularly effective in producing suspended droplets of liquids with low surface tensions [22]. Following the superhydrophobic literature, we can also anticipate that if the surface chemistry tends towards hydrophilic (i.e., θ_e_ < 90°) there might be a hemi-wicking effect with the liquid invading the surface texture, but wetting the asperities of the topographic features. A simple two-dimensional model consideration of the energy changes as a liquid invades a structure on a thin substrate suggests that the critical Young’s law contact angle for hemi-wicking will be shifted to values lower than θ_c_ due to the contribution of bending energy.

#### 1.4 Drop size and contact angle effects

The inclusion of the energy associated with the curvature of a substrate introduces a characteristic elastocapillary length and results in drop size effects. For a ribbon film substrate, Equation 10 implies wrapping requires the droplet radius *R* to be greater than a critical radius, *R*_c_, given by,

[15]
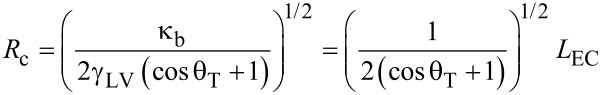


which can be compared to the condition *R* > *L*_EC_/√2 given by Py et al [3]. Thus, there is a critical radius which depends on the Young’s law contact angle, θ_e_, and the topographic structure via the surface roughness, *r*_W_, or solid surface fraction, φ_s_.

In the Cassie–Baxter case, cosθ_T_ = cosθ_CB_, and θ_CB_ can approach 180° from below and, as it does so, the critical radius for wrapping tends to infinity; a strongly superhydrophobic ribbon will not result in droplet wrapping because the energy gain cannot overcome the bending energy. In the Wenzel case, cosθ_T_ = cosθ_W_, and this is positive when θ_e_ < 90°, but negative when θ_e_ > 90°. In the former case, the critical radius becomes smaller as the Young’s law contact angle tends to zero or as the roughness increases; a film can be wrapped in a tighter curve and, hence, a smaller droplet radius is needed. It should also be noted that cosθ_e_ is defined by a combination of the interfacial tensions and this combination can be greater than unity; this corresponds to a film of liquid on a smooth and rigidly flat surface. In the considerations above, no account has been taken of the finite mass of the substrate on the critical volume of liquid required for wrapping; a problem recently considered experimentally and theoretically for square and triangular sheets of PDMS by Chen et al [23].

### 2. Liquid marbles and topographically structured grains

**Figure 4:**
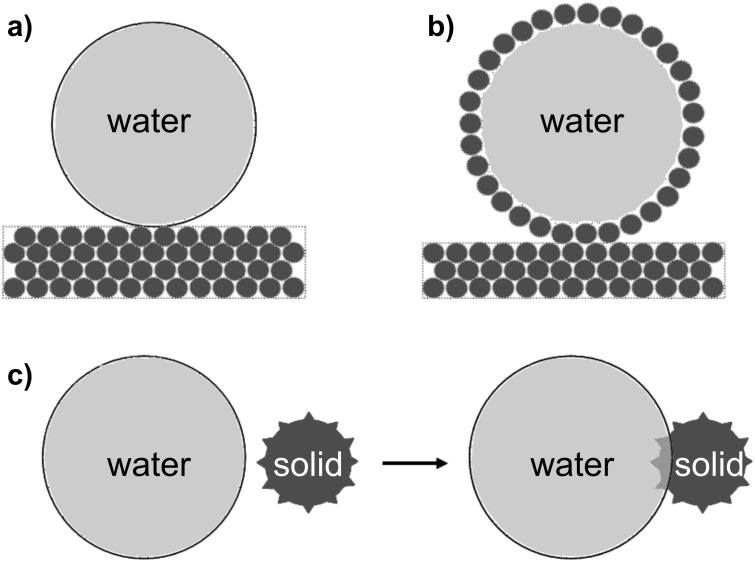
Formation of a liquid marbles: a) droplet contacting substrate composed of loose grains, b) attachment of grains to encapsulate a droplet, c) minimisation of surface free energy by replacement of a portion of the liquid–vapor interface by a portion of the rough solid surface from an attaching grain.

When a solid in the form of a thin ribbon wraps around a droplet it only needs to bend, whereas when the solid is a sheet it needs to either stretch and deform or to crumple and fold. Such a situation could be considered, but additional energies relating to these effects would need to be included unless the contribution from these is at no energy cost. One possible situation that conceptually is similar to a substrate able to deform and conform to a liquid surface, but without any bending or stretching energy cost, is the adhesion of a collection of solid grains to a liquid surface to encapsulate it and form a liquid marble (Figure 4a and Figure 4b) [12–13,24]. In an abstract sense, a collection of grains assembled in a close-packed form onto a liquid–vapor interface is the extreme limit of a flexible solid possessing no curvature (or stretching) energy and, hence, a vanishing elastocapillary length. In the study of liquid marbles, the simplest assumption is that each grain is spherical in shape and has no particular surface topography. As a consequence all grains, irrespective of their surface chemistry, will adhere to the water-air interface; a similar conclusion to that regarding the absolute hydrophilicity of solids when their curvature energy is zero. The effect of surface chemistry, characterised through the Young’s law contact angle, is to determine the strength of the adhesion to the air-water interface with maximum strength corresponding to θ_e_ = 90°; if θ_e_ > 90° more than half the grain projects out of the interface into the air. In practice, the surfaces of the grains do not need to be smooth and can have a topographic structure. For example, pollen grains come in a variety of shapes, commonly spherical, ovoid or disc-like with lengths in the order of 10–100 μm and their surfaces (exine) under scanning electron microscopy vary from relatively smooth to mesh-like and ones adorned with sharp spikes (see, e.g., [25]).

By considering the changes in interfacial areas as a spherical grain of radius *R*_g_ with a roughness *r*_W_ attaches to a droplet of radius *R* (Figure 4c), we deduce the change in surface free energy Δ*F*^M^_T_,

[16]
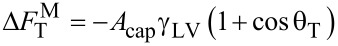


where *A*_cap_ = π*R*_g_^2^(1 + cosθ_T_) is the spherical cap area of the solid grain of radius *R*_g_ intersecting the droplet and θ_T_ is either the Wenzel contact angle or the Cassie–Baxter contact angle, depending on whether the liquid penetrates between the topographic features on the surface of the grain or whether it bridges between the asperities (and is therefore only in contact with a fraction of the solid area, φ_s_). In a similar manner to droplet wrapping, Equation 14 defines a minimum Young’s law contact angle for the Cassie–Baxter state to be thermodynamically stable over the Wenzel state. The idea of a solid film that tends to a non-adhesive surface for liquids can be extended to non-stick granular or powder systems. All smooth spherical grains adhere to the liquid interface because (1 + cosθ_e_) can never be negative. However, when the surface of a grain is structured it can become superhydrophobic and it will then only weakly attach to the surface of the liquid.

## Conclusion

In this work, we have focused on a rigid surface structure on a thin flexible substrate, but the inverse situation of a flexible surface structure on a rigid substrate has recently also been modelled [26–27]. A result of that work is an understanding that elastocapillary effects can provide additional stability for Cassie-type suspended liquid states involved in, e.g., plastron respiration [27–29]. It therefore seems likely that to fully understand superhydrophobic surfaces, the flexible nature of elements of surfaces needs to be understood. Using a model of a thin ribbon (strip) substrate we have shown that relaxing the assumption of a rigid substrate allows a contacting droplet to shape and bend the substrate provided the droplet radius is larger than a critical value. When the flexible substrate has a surface with a rigid topographic structure, the critical droplet radius at which droplets wrap depends on both the elastocapillary length and a function of either the Wenzel or the Cassie–Baxter contact angle dependent on the state of the contact. We have argued that liquid marbles can be thought of as such a system, but with a vanishing elastocapillary length. Manipulating the surface structure therefore provides a method, complementary to control of substrate thickness, to tune the balance of adhesive forces between liquids and solids both within *capillary origami* and granular systems.
